# Examination of Gender Difference in Heart Disease-Related Excess Deaths during COVID-19 Pandemic Era: Findings from the United States

**DOI:** 10.31083/j.rcm2305182

**Published:** 2022-05-18

**Authors:** Hang Nguyen, Haekyung Jeon-Slaughter

**Affiliations:** ^1^Department of Statistical Science, Southern Methodist University, Dallas, TX 75205, USA; ^2^VA North Texas Health Care System and Department of Internal Medicine, University of Texas Southwestern Medical Center, Dallas, TX 75390, USA

**Keywords:** COVID-19, non-COVID-19 excess deaths, heart disease-related mortality, gender, cause of death

## Abstract

**Background/Objective:**

Heart disease is the leading cause of death among women in the United States, and women are experiencing more strokes at younger ages than men. Despite accumulating evidence of increased burden of heart disease among women, there is little data on gender difference in heart disease-related mortality during the COVID-19 pandemic.

**Method:**

This study extracted the data of weekly number of deaths between January 2017 and December 2020 from the United States Center for Disease and Control and Prevention (CDC) mortality and morbidity data, modified to a monthly scale. Stratified by gender, the study applied the Farrington method on monthly data to calculate excess number of deaths. Excess heart disease-related deaths were observed in March and July 2020 for both males and females.

**Results:**

While the overall number of heart disease-related deaths was higher in men than women among US population <75 years old, a greater rate increase of heart disease-related deaths in 2020 from 2019 was observed among women than men. This increased burden was more pronounced among young women <25 years old. A similar pattern of excess deaths caused by underlying heart disease condition was observed for both genders during COVID-19 pandemic. On the other hand, increase in heart disease-related death burden in 2020 from 2019 was greater amongst females than males. This may be partially accounted for by deferred cardiovascular care and prevention amongst women during the pandemic.

**Conclusions:**

While no gender difference was observed in excess deaths caused by underlying heart disease condition, females faced a greater increase in heart disease-related death burden during the pandemic compared to pre-pandemic than males.

## 1. Introduction

The United States (US) experienced over a half million excess deaths one year after the COVID-19 pandemic started [1, 2]. While 72% of these deaths were attributed to COVID-19, the rest were non-COVID disease-related. The leading non-COVID-19 cause of excess deaths was heart disease in 2020. These non-COVID-19, heart disease-related excess deaths are partly due to disrupted or deferred care [3]. The excess deaths since March 2020 were a minimum of 330,000, and a maximum of 412,000, and about 67% of excess deaths were attributed to COVID-19 and its complications as of January 03, 2021 [2].

Almost one third of excess deaths were attributed to non-COVID related causes such as delayed and deferred care during the COVID-19 pandemic [1]. Some research reported that patients who had heart attacks or strokes delayed seeking care due to fear of COVID-19 infection at hospitals during the pandemic period [4].

Heart disease is the leading cause of death among women, and women are experiencing more strokes at younger ages than men. Approximately 300,000 women died of heart disease in the Unites States in 2017 alone [5]. More studies showed that young women age under 45 were at much higher risk of cardiovascular disease than previously thought [6] and experienced more strokes than their male counterparts at younger ages (under 45) [7]. A large US cohort study reported 17 strokes per 100,000 young women aged 25–34 compared to 12 strokes per 100,000 among their male counterparts [8].

While the number of heart disease-related deaths among males was higher than females, heart disease was the leading cause of death among females before the COVID-19 pandemic. Studies with early pandemic data reported that a male gender is associated with higher all-cause mortality [9], however there is accumulating evidence that disease burden may have been disproportionally increased among women with lower socio-economic background during the COVID-19 pandemic [10]. The literature showed that middle-age women may have been affected by burdens from family responsibilities and job loss, thus psychological tolls and mental stress from COVID-19 may be greater among women than men [11, 12]. It is known that psychological stress affects heart health [13].

Despite accumulating evidence of increased burden in heart disease among women, there is a lack of studies investigating gender difference in heart disease burden during the COVID-19 pandemic. The study examines whether there is a gender difference in heart disease-related mortality burden during the COVID-19 pandemic using US Center for Disease Prevention and Control (CDC) mortality count data between January 01, 2017 and December 31, 2020.

## 2. Results

Table 1 shows the crude heart disease-related death rate per 100,000 by age group, stratified by gender. Crude death rates in 2020 were higher compared to 2019 for both genders with crude rates higher among males than females in both years. The crude heart disease-related death rates increased exponentially with aging starting from age 65 in both 2019 and 2020 across genders.

**Table 1.** **Heart disease-related crude death rate per 100,000 by gender and age group for 2019–2020**.

2019		Under 25	25–44	45–64	65–74	75–84	85+	Total
	Male	0.93	21.1	191.5	537.2	1261.2	4302.4	221.0
	Female	0.47	9.7	79.6	258.8	858.5	3515.0	188.0
2020								
	Male	0.85	24.1	205.6	567.7	1348.8	4797.7	232.3
	Female	0.52	11.5	86.3	281.0	833.6	3864.5	187.1

Note. (1) Crude rate per 100,000 [(Number of deaths/Total population per age group) × 100,000].

**Fig. 1.**
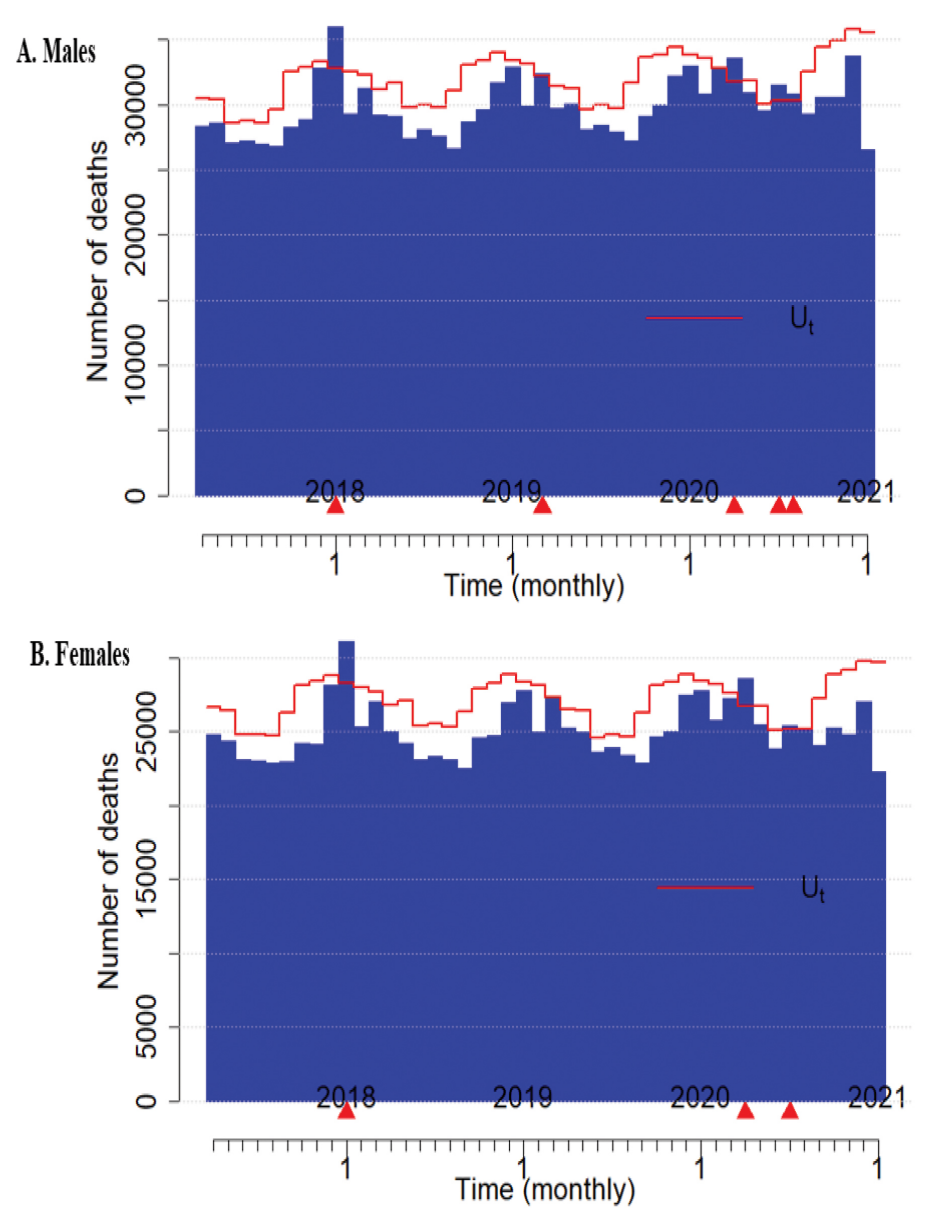
** Excess heart disease-related deaths in 2017–2020 by gender**. (1) Upper panel is Males and B is Females. (2) A red line represents upper bound of predicted number and blue bars observed numbers. Excess is observed when the observe numbers are greater than the upper bound of predicted numbers. (3) Red bar portions indicate excess numbers and red triangles point out months with excess numbers. In the horizontal line time month “1” indicates January of each year and a grid represents a month period. (4) For males (upper panel A), excess numbers of heart disease-related deaths were observed in March, June, and July 2020 during the COVID-19 pandemic. For females (lower panel B), excess numbers of heart disease deaths were observed in March 2020 and June 2020.

Excess numbers of heart disease-related deaths were observed in March and June 2020 for both genders and in July for males only (Fig. 1).

Fig. 1 shows excess numbers of heart disease-related deaths between January 01, 2017 and December 31, 2020. For males, excess numbers of heart disease-related deaths were observed in March, June, and July 2020 during the COVID-19 pandemic. For females, excess numbers of heart disease-related deaths were observed in March 2020 and June 2020. Also, there were excess number of heart disease-related deaths pre COVID-19. For both genders, there was an excess number of heart disease-related deaths in January 2018. For males only, there was another excess number of heart disease-related deaths in February, 2019.

### 2.1 Gender Difference in Aging Trajectory of Heart Disease-Related Deaths 

Overall, a significantly higher number of males died from heart disease during the COVID-19 pandemic than females among age groups under 85 with a linear aging trajectory. On the other hand, the absolute number of females that died from heart disease is greater than males among age group 85+.

Number of heart disease-related deaths among both genders jumped at age 45 and then increased linearly with aging. Notably, the number of heart disease-related deaths among women aged over 85 surpassed their male counterparts.

### 2.2 Gender Difference in a Trend of Heart Disease-Related Deaths between 2017 and 2020

Overall, the number of heart disease-related deaths was largest in year 2020 among males across all age groups. This was true for females except age group of 75 and older, whose trend differed from the rest of gender and age groups. Unlike other groups, number of heart disease-related deaths in 2020 among female aged 75–84 was smaller in 2020 than 2019, while stays the same for a female age group of 85+. When considering only age groups between 25 and 64, we observed a slight downward trend in number of heart disease-related deaths from 2017 until 2019, but this downward trend was reversed in 2020, with a sharp increase for both genders.

### 2.3 Gender Difference in Change of Heart Disease-Related Mortality Rate in 2020 from 2019

Table 2 shows changes in rates in 2020 from 2019 by age group per gender. In 2020, heart disease-related crude mortality rates for both genders increased from 2019 among all age groups of 25 and plus, except a female age group of 75–84 (Table 1). This rate increase was higher in females than males among the age groups of <75. In particular, this observation was more pronounced among females under 25 years old (Table 2). While heart disease-related death rate for young males under 25 decreased by 8.2% in 2020 from 2019, it increased by 9.4% among their female counterparts. The change in heart disease-related death rate in 2020 from 2019 was the highest among younger women aged between 25 and 44, and larger than their male counterparts.

**Table 2.** **Percent of change in 2020 mortality rate from 2019 by age group for each gender**.

	Under 25	25–44	45–64	65–74	75–84	85+
Males	–8.23	14.32	7.36	5.67	6.94	11.51
Females	9.43	18.67	8.41	8.53	–2.91	9.94

Note. (1) Percentage change in mortality = [(mortality rate in 2020 – mortality in 2019)/mortality in 2019) × 100].

For the age groups of 75 plus only, heart disease-related crude death rates increased more in males than females in 2020 from 2019. US males’ heart disease-related death rate increased in 2020 from 2019, while it decreased among US female age group 75–84. Overall increased crude mortality rate in 2020 from 2019 among the male age group of 85 plus was greater than their female counterparts.

## 3. Discussion

During the COVID-19 pandemic, we observed a similar pattern of excess deaths caused by underlying heart disease condition for both genders. While the overall number of deaths was still greater in males than females, we found data evidence that heart disease-related deaths had a disproportionately greater impact on young (age <25) and younger (ages 25–44) female population than their male counterparts. A greater rate increase of heart disease-related deaths in 2020 from 2019 was observed among women than men, especially, young and middle-age groups.

This observed trend raises concerns because women under age 45 are believed to be at no or a minimum risk of cardiovascular disease (CVD) events according to the current CVD care guideline. Increased heart disease-related mortality rate observed during 2020 is partly due to delayed or deferred cardiovascular care during pandemic but also may reflect a missed opportunity of prevention at an earlier stage of CVD.

The study findings may indicate that demographic groups affected by non-COVID-19-related deaths during the pandemic era may differ from groups most affected by COVID-19 itself. Compared to COVID-19 associated mortality risk factors—older age [14, 15] and male gender [9, 14] were significantly associated with increased risk of COVID-19-associated mortality after infection, increased risk of dying from non-COVID-19 disease such as heart disease during the pandemic era, may be associated with different age and gender groups, such as younger females.

The study finding—increased risk of dying from heart disease among younger population during COVID-19 pandemic era, is also consistent with a previous study using US state of Texas data of age groups 25 and 44 [16]. The previous study [16] reported that heart disease was a third leading cause of death next to accidents and assaults without stratifying by gender during the first year of the COVID-19 pandemic from March 2020 to December 2020.

The current study found not only a similar excess death caused by heart disease conditions between two genders, but also found a pronounced gender disparity in change on heart disease-related death burden in 2020 compared to 2019 among the younger females under age 45.

To note, an excess number of heart disease-related deaths was also observed in the US pre COVID-19 era. The study results showed an excess number of deaths in January 2018 for both genders. This is partly due to a fact that US had one of highest mortality rate during a seasonal flu and its complications during the winter of 2018 according to the CDC [17].

Our study finding is also consistent with US excess deaths in non-COVID-19 heart disease mortality during the COVID pandemic era reported in the previous studies [1, 2]. In addition, it presents new and important information on gender difference in heart disease-related mortality by age group during the pandemic. This study finding highlights an increased gender differential in heart disease-related mortality burden among the younger US population during the COVID pandemic.

This may be partly due to younger women being more likely to defer preventive and routine care during the pandemic because of increased burden of caregiving to family members. Women are more likely to be unpaid caregivers to family members during the COVID pandemic [18], and unpaid caregivers were more likely to defer a necessary care during the pandemic [19]. Furthermore, young women are underestimated for cardiovascular disease risk under the current guideline [6, 20], thus they were more likely to be under-treated for underlying heart disease conditions pre pandemic, which was exacerbated by deferring care during the pandemic [18, 19].

The study found that number of heart disease-related deaths among women aged ≥85 was greater than their male counterparts, which differs from other countries [19, 21]. While underlying cause of this difference is unknown, Islam *et al*. [21] provided some explanations. Overall, the demographic group of age over 85 was the most affected age group by mortality during the COVID pandemic. The number of US female population older than 85 in 2019 was much larger than their male counterparts, 178 women to 100 men according to US Department of Human Services [22]. In addition, heart disease is the leading cause of death among older female US population. These may account for a higher number of heart disease-related deaths observed among women over 85 than their male counterparts in the US compared to other countries, despite the opposite being true for all other age groups among US population.

Cardiovascular complications are commonly reported among those who recovered from COVID-19, and thus, COVID-19 patients may have shown a higher rate of cardiovascular disease prevalence even amongst those who recovered from COVID-19 [23, 24, 25, 26]. Number of patients with heart disease as the reported cause of death in death certificates may include those who recovered from COVID-19 but died from cardiovascular complications of the COVID-19 infection. Thus, the increase in crude heart disease-related death rates in 2020 from 2019 can be partly accounted for by high prevalence of cardiovascular complications among patients who were recovered from COVID-19. The statistical method used in the study, Farrington Algorithm, is the most accepted method in estimating excess deaths related to disease outbreak [1, 2, 27], however, there are also other existing methods to estimate excess deaths. Islam *et al*. [21] used a parametric modeling approach, e.g., Poisson model, to predict mortality, while Faust *et al*. [16] used time series analysis—autoregressive integrated moving averaging method. The Farrington algorithm used in the current study focused on detecting a short period, spiked number of deaths related to surges of COVID-19 cases, while other methods such as times series analysis focused on estimating a sustained trend in death counts. The COVID-19 pandemic data follows more closely to disease outbreak model. Thus, Farrington algorithm is an appropriate method to estimate excess deaths specific to heart disease during the COVID-19 pandemic [28].

The study has limitations. The study data analysis focused on univariate temporal analysis due to a nature of the study data—monthly count data. The current study data, counts data limited to gender stratification, do not include other demographic factors, such as a geographic location and race and ethnicity, potentially associated with excess deaths. However, the aim of the study is to investigate whether there is a gender difference in deviation from a trend in heart disease-related death during COVID-19 pandemic. Also, we cannot eliminate a possibility that heart disease as cause of death may be under-counted due to missing data on cause of death or under-reported due to delay of surveillance data input. The data used in this study was monthly count data due to a small number of weekly count data.

Another limitation of the current study is a lack of information on the subtypes of heart disease as cause of death. Mortality burdens of hypertensive heart disease, acute myocardial infarction, and acute heart failure have shown different trends from each other during the pre COVID-19 period [29, 30]. Thus, a pattern of excess deaths during the COVID-19 pandemic could also be different by the subtypes of heart disease.

Future studies are needed to confirm a relationship between the gender difference in deferring care and the increased gender disparity in heart disease-related mortality during the COVID-19 pandemic.

## 4. Conclusions

In conclusion, despite that overall number of heart disease-related deaths was higher in men than women among US population younger than age 75, COVID-19 impact on increase in heart disease-related mortality burden was more pronounced among women, particularly women younger than 25 years old, than men. With the COVID-19 pandemic still at large at the time of the current study, deferring care for cardiovascular disease and prevention among younger women will continue, thus a gender disparity in non-COVID-19 disease burden such as heart disease expects to be further widened.

## 5. Materials and Methods

The study extracted data about weekly number of deaths, between January 01, 2017 and December 31, 2020 from the US CDC website and converted to monthly number of deaths. The CDC mortality counts data are based on information collected from death certificates from 50 US states and District of Columbia. The mortality data then be complied to the national data by the National Center for Health Statistics (NCHS) [31]. The national mortality counts are available as weekly counts and also stratified by gender.

The study compiled daily and weekly data to monthly counts of heart disease-related deaths from January 01, 2017 until December 31, 2020. The study identified heart disease-related deaths using cause of death from CDC mortality data. Cause of death is specified by disease which is coded using International Classification of Disease (ICD) 10 codes. Corresponding ICD-10 codes for heart disease are I00–I09, I11, I13, and I20–I51 [27, 32]. Stratified by gender, the study followed the Farrington method to calculate excess number of deaths using modified monthly data [33, 34].

Following Farrington algorithm, we calculated upper bounds of expected or predicted monthly numbers of heart disease-related death incidents, and the difference between the observed numbers of incidents and upper bounds. When observed numbers were greater than the upper bound expected or predicted numbers, defined as excess death. The Farrington algorithm intended to detect aberration on an univariate time series of counts t = 1,2,…T, interpreted as an unexpected surge or change in trend, occurring at time t. The 95% confidence intervals (CI) of estimated predicted excess deaths were computed by over-dispersed Poison generalized linear model with log link. The upper bound of 95% CI was estimated as a quantile of the negative binomial distribution with the corresponding estimated mean and variance.

The study used “Surveillance” package available from R statistical program (https://cran.r-project.org/) and applied modified Farrington algorithm [34] to monthly aggregate data to detect any unusual and unexpected trend of heart disease-related mortality during the study period.

Descriptive statistics and graphs were presented to describe a gender difference in numbers, rates, and change in rates heart disease-related mortality. All statistical and graphic analyses were conducted by R (https://cran.r-project.org/).
